# Mechanistic Links Between Obesity and Breast Cancer Progression: Cellular Crosstalk, Metabolic Reprogramming and Microenvironmental Drivers

**DOI:** 10.1002/cbf.70263

**Published:** 2026-07-13

**Authors:** Charlise Basson, Michael S. Pepper, Anna M. Joubert, Melvin A. Ambele

**Affiliations:** ^1^ Department of Oral and Maxillofacial Pathology, School of Dentistry, Faculty of Health Sciences University of Pretoria Pretoria South Africa; ^2^ Department of Medical Immunology, Institute for Cellular and Molecular Medicine, and SAMRC Extramural Unit for Stem Cell Research and Therapy, Faculty of Health Sciences University of Pretoria Pretoria South Africa; ^3^ Department of Physiology, School of Medicine, Faculty of Health Sciences University of Pretoria Pretoria South Africa

**Keywords:** adipokines, adipose tissue, breast cancer, cancer metabolism, obesity, oxidative stress, tumour microenvironment

## Abstract

Obesity is increasingly recognised as an important factor contributing to cancer progression, particularly in breast cancer. However, the cellular and molecular mechanisms underlying obesity‐driven breast cancer remain unclear. Several theories have been proposed to explain the complex interactions between adipose tissue and cancer, incorporating both local and distant crosstalk. This review describes existing theories on the cellular and molecular mechanisms linking obesity and breast cancer progression with a focus on inflammation, oxidative stress, metabolic reprogramming, the tumour microenvironment and crosstalk between adipose tissue and tumours. The nature of these interactions appears to be influenced by the specific characteristics of adipose tissue, including its type and anatomical location, which play distinct roles in modulating breast cancer risk and outcomes. Understanding the cellular and molecular mechanisms that underlie obesity‐driven breast cancer progression may pave the way for the development of targeted therapies.

## Summary

Obesity is a major modifiable risk factor for breast cancer incidence, progression and metastasis. Despite the reported association between obesity and poor breast cancer outcomes, the exact underlying biological mechanisms remain incompletely defined. Theories on the cellular and molecular mechanisms underlying the cancer–obesity relationship include direct adipose–tumour interactions, inflammation, oxidative stress and metabolic reprogramming. Understanding how obesity‐associated factors influence breast cancer is essential for improving current therapies and identifying new therapeutic targets in patients with obesity.

## 1 Introduction

In addition to its well‐established effects on metabolic and general health, obesity is known to contribute to a number of malignancies, including breast cancer [1]. With both breast cancer mortality rates [2] and obesity incidence predicted to continue rising [3], there is an urgent need to understand the relationship between obesity and breast cancer. Obesity is believed to play a role in the incidence and progression of breast cancer [4]. However, the precise pathophysiological mechanisms by which obesity contributes to breast cancer initiation, progression and metastasis remain largely unclear [5].

Multiple theories have been proposed to explain the mechanisms by which obesity drives breast cancer progression. These theories focus on adipose tissue‐dependent secretion of molecules that may directly affect primary breast cancer tumours, alter the tumour microenvironment (TME), or prime secondary metastatic sites. These mechanisms are thought to be controlled by the adipose tissue‐dependent secretion of bioactive molecules, such as adipokines, cytokines and chemokines. However, these factors induce distinct effects based on their location and the type of adipose tissue from which they are secreted [6].

This review explores the current understanding of the molecular and microenvironmental mechanisms linking adiposity to cancer progression, highlighting the roles of inflammation, oxidative stress, metabolic plasticity and adipose–tumour interactions in shaping tumour behaviour. This knowledge may inform targeted therapeutic strategies and is necessary for implementing effective public health interventions aimed at reducing the obesity‐related cancer burden.

## 2 Methodology

This narrative review used three databases: PubMed, Scopus and Web of Science. The search strategy included a combination of Medical Subject Headings (MeSH) terms, with the Boolean operators ‘AND’ and ‘OR’ used for relevant topics discussed in the review. The inclusion criteria were articles published in English from 2015 to 2025. This time frame was chosen to capture the most recent advances in knowledge of the cellular and molecular underpinnings of obesity‐driven breast cancer. However, foundational studies prior to 2015 were strategically included, where conceptual clarity was necessary to support key findings. Mechanistic (in vivo and in vitro), animal and clinical studies were included, given that few studies comprehensively investigate the underlying mechanisms of obesity‐driven breast cancer and often evaluate only individual components rather than a full mechanistic framework or system. Each study was screened individually, and non‐English articles and studies with irrelevant diseases and/or treatment contexts were excluded. The included articles were exported to EndNote (version 20) reference manager.

## 3 Adipose Tissue: Type and Location Matter

The presence of heterogeneous adipose tissue in various depots, along with its numerous functions, complicates the understanding of the role of adipose tissue in cancer [7]. Adipose tissue has traditionally been broadly classified into white adipose tissue (WAT) and brown adipose tissue (BAT). Additional variants, such as beige and pink adipose tissue, have recently been identified [8]. However, the development, metabolism, homoeostasis, and cellular progenitors of the different types of adipose tissue remain incompletely understood [9]. As such, its effects on cancer have been described in different contexts in cancer, which is summarised in Table 1.

**Table 1:** Effects of adipose tissue types on cancer.

Type of adipose tissue	Characteristics	Location	Biological role	Potential role in breast cancer				References
				Advantage	Evidence	Disadvantage	Evidence	
Brown adipose tissue (BAT)	Brown ↑ Iron‐rich mitochondria Secretes batokines ↑ UCP1	Paravertebral region Smaller amounts found in axillary, cervical, perivascular and perirenal regions	Anti‐inflammatory Improve insulin sensitivity Enhance thermogenesis ↓ Obesity risk	Possibly decrease cancer risk due to an improvement of systemic metabolism, inflammation and insulin activity	Breast cancer patients with BAT activity have shown longer progression‐free survival BAT activation (through cold exposure) reduced tumour growth, hypoxia, angiogenesis, CD45^+^ myeloid cells and increased survival of tumour‐bearing mice Patients with BAT activation had a smaller primary tumour size	↑ Overall energy expenditure, which could lead to cachexia	Patients with higher BAT volume showed increased tumour‐associated mortality	[7, 10, 11, 12, 13, 14, 15, 16, 17, 18, 19, 20, 21, 22, 23]
Beige adipose tissue	Beige UCP1‐dependent or independent	WAT depots	WAT transitions to a thermogenic state for heat production during cold exposure Changes are reversible upon exposure to heat	Protection against obesity	WAT beiging/browning occurred, even in the absence of BAT during cold exposure, highlighting beige adipocytes as promising targets for obesity	This state might precede cancer cachexia, have no risk or increase cancer cell aggressiveness	WAT browning precedes the loss of muscle in the early phases of cancer cachexia No association between BAT activation and cachexia or increased mortality in patients with cachexia after controlling for confounding factors No correlation between changes in cancer burden and BAT activity or BMI Cancer cells exposed to beige adipocyte conditioned media displayed increased aggressiveness through migration and invasion	[7, 12, 19, 24, 25, 26, 27, 28, 29, 30, 31]
Pink adipose tissue	Pink Subcutaneous	Mammary adipose tissue	During pregnancy, lactation and post‐lactation	↑ Cancer aggressiveness	Mammary adipose stromal cell populations present during lactation (but not during other mammary developmental stages) promoted breast tumour growth (3.5 fold) in a syngeneic in vivo mouse model Mammary gland involution promoted breast tumour growth in a murine model	Limited evidence available	Limited evidence available	[32, 33, 34]
White adipose tissue (WAT)	White	Subcutaneous fat (SCAT), typically found beneath the skin (the femerogluteal regions, back and anterior abdominal wall) Visceral adipose tissue (VAT), around internal organs (mesentery and omentum)	Secretes adipokines, leading to metabolic dysfunction and inflammation	↑ Cancer aggressiveness	Higher VAT correlates with worse outcomes in patients with metastatic colorectal cancer, endometrial cancer and breast cancer	Higher VAT and cancer aggressiveness might be context (cancer and metabolic status) dependent	VAT previously predicted better survival in men but worse in women with rectal cancer	[35, 36, 37, 38, 39, 40, 41]

### 3.1 Brown Adipose Tissue

BAT is characterised by its brown colour, which arises from its high content of iron‐rich mitochondria [10]. It is primarily located in the paravertebral region, with smaller amounts found in axillary, cervical, perivascular and perirenal regions [7]. The primary function of BAT is to regulate thermogenesis (the production of heat), which in turn is regulated by expression of uncoupling protein 1 (UCP1), which enables mitochondria to dissipate energy as heat instead of producing ATP [7].

BAT also secretes signalling molecules, including peptides and metabolites, collectively termed batokines, which influence various physiological processes, such as the regulation of metabolism, thermogenesis and systemic energy homoeostasis through autocrine, paracrine and potentially endocrine mechanisms. The term mirrors the concept of ‘adipokines’, commonly associated with WAT, but applies specifically to factors released by brown adipocytes [42]. A previous study of the human BAT secretome identified 471 proteins, of which 101 were found to be uniquely secreted by BAT compared to WAT [43]. Batokines, such as neurotrophin 3 (NT‐3), bone morphogenetic protein 7 (BMP‐7), bone morphogenetic protein‐8b (BMP8b), chemerin, follistatin‐like 1 (FSTL1), fibroblast growth factor 21 (FGF21), neuregulin 4 (NRG4), irisin, nesfatin‐1, meteorin‐like protein (METRNL), vascular endothelial growth factor A (VEGF‐A), interleukin (IL)−6, IL‐8 and IL‐10 exert effects on thermogenesis, glucose homoeostasis, insulin sensitivity, energy expenditure, lipid metabolism, angiogenesis and anti‐inflammation [11, 12], which are all believed to counteract breast cancer progression.

BAT has been demonstrated to have a protective effect against metabolic dysfunction by clearing acylcarnitines, triglyceride‐rich lipoproteins, glucose and branched‐chain amino acids, leading to the improvement of systemic metabolism, inflammation and insulin activity [7]. As such, BAT demonstrates significant potential in promoting metabolic health and opposing obesity‐related conditions. An increase in BAT fosters an anti‐inflammatory environment, supporting healthy tissue development, improving insulin sensitivity, enhancing thermogenesis and ultimately reducing obesity. These metabolic improvements may indirectly reduce the risk of cancer [13]. This is of particular importance, as chronic hyperinsulinemia and inflammation promote cancer progression by activating cellular signalling pathways leading to enhanced proliferation and metabolic changes that support tumour growth [14, 15].

It has been demonstrated that cold‐induced BAT activation inhibited tumour growth, hypoxia and angiogenesis, reduced CD45^+^ myeloid cell populations and increased the survival in tumour‐bearing mice. Mechanistically, these anti‐cancer effects may have been attributed to the metabolic reprogramming of tumours by suppressing glucose metabolism (glycolysis) [44]. BAT activity, as observed using 18F‐fluorodeoxyglucose (FDG)‐Positron Emission Tomography (PET)/Computed Tomography (CT) scans, was previously shown to be three times more prevalent in patients with breast cancer than in age‐ and weight‐matched patients with other solid tumours [45]. Additionally, patients with breast cancer with BAT activity have shown longer progression‐free survival than those without [46]. These results were confirmed in a study showing that patients with BAT activation had a smaller primary tumour size. Additionally, BAT activation was associated with younger age (< 50 years), lower outdoor temperature and lower BMI [47]. In a breast cancer patient cohort, BAT activation decreased after a single course of chemotherapy and patients who demonstrated the greatest decrease in BAT activity were also the patients who gained > 5% weight [48].

Conversely, a longitudinal study by Chu and colleagues showed that a higher BAT volume correlated with an increased tumour‐associated mortality [16]. One explanation for these opposing effects of BAT could be tumour‐ and context‐dependent, and might differ when comparing cross‐sectional to longitudinal studies. Additionally, the higher BAT volume observed in patients with cancer may induce cancer cachexia, which could contribute to cancer mortality. Huang and colleagues demonstrated that patients with a history of cancer had a wider BAT distribution and higher total metabolic activity [17]. Indeed, WAT browning leads to lipolysis and ultimately results in increased energy expenditure [18], which may play a role in pathological conditions such as cancer cachexia [19, 20], that is inversely associated with survival and quality of life [21]. Cancer‐associated cachexia is a multifactorial syndrome resulting in wasting of several tissues, including skeletal muscle, cardiac muscle, liver, bone, the gastrointestinal tract and adipose tissue [22]. A systematic review and meta‐analysis reported that women with breast cancer have a significant prevalence of sarcopenia (muscle loss), which is often present in patients with cachexia (muscle and weight loss) [23].

### 3.2 Beige Adipose Tissue

A third type of adipocyte has been described, which is neither white nor brown but is described as brown‐in‐white or beige. These beige adipocytes originate from a lineage more closely related to white adipocytes and reside in WAT depots [24]. During cold exposure or heightened adrenergic stimulation, WAT can transdifferentiate from white to brown (transition into a thermogenic state) in a process called ‘beiging’ or ‘browning’, giving rise to beige adipocytes, also referred to as ‘brite’ adipocytes [25]. Transdifferentiation refers to the process by which a mature, differentiated cell transitions into a distinct cell phenotype characterised by different morphology and physiological functions, without first dedifferentiating [49]. Beige adipocytes typically display low UCP1 expression, but upon cold exposure they undergo changes in cell structure, allowing them to increase UCP1 expression (similar to BAT) [28, 50]. However, beige fat cells are unique in that they can also utilise UCP1‐independent mechanisms, including futile creatine and calcium cycling for heat production [26]. It should be noted that the changes induced by browning are reversible and regress when the environment heats up again [7]. Nevertheless, beiging/browning has been hypothesised as a potentially promising therapeutic approach in obesity due to its thermogenic effects [50]. This has been shown in a previous study where WAT beiging/browning occurred, even in the absence of BAT during cold exposure. As such, beige adipocytes were highlighted as important regulators of systemic metabolism and promising targets for obesity [27].

**Figure 1:**
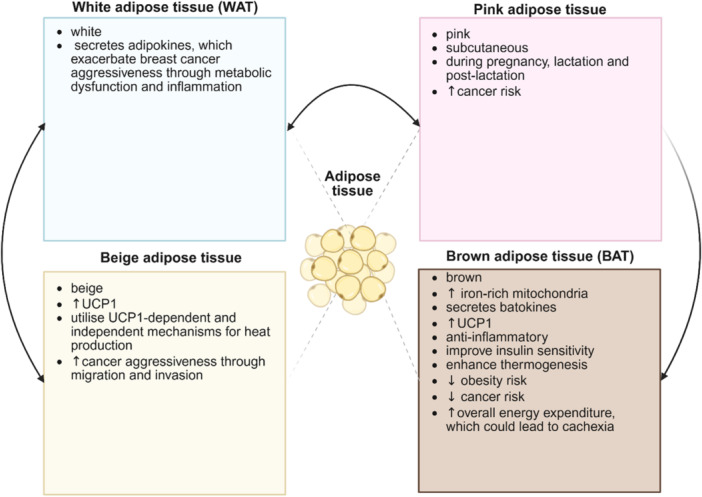
The role of various adipose tissue types, including WAT, BAT, beige and pink adipose tissue in breast cancer. Different types of adipose tissues may have distinct effects on breast cancer progression. Additionally, the interconversion between these adipose types (indicated by arrows) highlights the plasticity of adipose tissue in shaping the TME of tumours. This figure was created with BioRender.com.

However, in the context of cancer, it is mediated by pro‐inflammatory mediators, which may be tumour‐derived or catecholamines during cachexia [12]. As such, beiging/browning may represent a paradox in cancer progression, by offering protection against obesity‐driven cancer progression while inducing cachexia. Increasing evidence indicates that WAT browning precedes the loss of muscle in the early phases of cancer cachexia [19]. Additionally, the inhibition of WAT browning may lessen the severity of cancer cachexia [28], which accounts for 20% of all cancer‐related deaths [51]. However, contrasting evidence suggests that there is no association between BAT activation and cachexia or increased mortality in patients with cachexia after controlling for confounding factors [29]. Similarly, a retrospective, longitudinal analysis of PET scans from 283 patients with various levels of tumour burden, showed no correlation between changes in cancer burden and BAT activity or BMI (Figure 1) [30].

Through their secreted factors, beige adipocytes can significantly alter the adhesion, migration and molecular profile of breast cancer cells, supporting a pro‐metastatic phenotype. This was demonstrated in a study in which mammary epithelial cells were exposed to conditioned media from beige or white adipocytes. Both cancerous (LM3, 4T1 and MC4‑L1) and non‐cancerous (NMuMG) mouse mammary epithelial cell lines exhibited reduced adhesion and increased migration. Cancer cells (MC4‐L1) exposed to beige adipocyte conditioned media also displayed upregulation of markers associated with aggressiveness, including ObR (leptin receptor) and MMP‐9 [52]. Similarly, another study found that beige adipocytes promote triple‐negative breast cancer (TNBC) progression through BMP4 signalling, which led to increased cancer cell migration and invasion [31]. These effects were also seen in vivo, where breast tumours consistently upregulated beige and BAT markers during progression, in both xenografts from three breast cancer cell lines and patient‐derived xenografts, regardless of implantation site (Figure 1) [53].

### 3.3 Pink Adipose Tissue

During pregnancy, lactation and post‐lactation, subcutaneous adipose tissue can transdifferentiate into specialised milk‐secreting alveolar cells named pink adipocytes [32]. These cells contain abundant cytoplasmic lipid droplets, an apical surface featuring microvilli, a centrally located large round nucleus, a rough endoplasmic reticulum, a Golgi complex and milk‐containing granules (Figure 1) [54].

The concept of plasticity in mammary adipose tissue was established in the early 2000s. In 2009, Cinti demonstrated reversible mammary adipocyte transdifferentiation in response to pregnancy hormonal stimuli [55]. Later, in 2014, a study by McCready and colleagues showed that mammary adipose stromal cell populations present during lactation (but not during other mammary developmental stages) promoted breast tumour growth (3.5 fold) in a syngeneic in vivo mouse model [33]. In 2018, Wang and colleagues further elaborated on the plasticity of mammary gland adipocytes by showing repeated cycles of de‐differentiation and re‐differentiation under various physiological conditions. In this study, mammary gland adipocytes were replaced by mammary alveolar structures during pregnancy and lactation, but reappeared upon weaning. These adipocytes de‐differentiated into Pdgfra+ preadipocytes and fibroblast‐like cells during pregnancy and remained de‐differentiated during lactation. However, dedifferentiated fibroblasts proliferated and re‐differentiated into adipocytes upon weaning. Notably, the authors highlighted the importance of future studies to investigate the effect of a high‐fat diet on these cycles of mammary gland de‐differentiation and re‐differentiation [56].

Other studies have specifically focused on mammary gland involution, which refers to the process after weaning, where the mammary gland regresses from a lactating to a non‐lactating state. In an early foundational study (2009), De Matteis and colleagues investigated the mechanisms that control changes in the size and behaviour of adipocytes and found that mammary gland‐associated WAT expands during involution due to adipocyte hypertrophy, which takes up milk‐derived lipids from epithelial cells. Additionally, normal milk trafficking from the epithelium and mammary gland remodelling was disrupted upon mammary gland‐associated WAT ablation [57]. Later, Martinson and colleagues showed that mammary gland involution promoted breast tumour growth in a murine model. Importantly, they showed that this could be attributed to increased IL‐10‐producing macrophages and Foxp3^+^ regulatory T cells, and reduced CD4^+^ and CD8^+^ T cell infiltration, which leads to immunosuppression [34]. Immunosuppression is also characteristic of an obesogenic state, which promotes cancer survival [58].

Although these studies display a potential link between mammary gland adipocytes and breast cancer, the underlying mechanisms involved and the influence of obesity on this link should be investigated [59]. To the best of our knowledge, the potential anti‐cancer effects of pink adipose tissue have not been reported. However, most research highlights persistent cycles of mammary adipose tissue differentiation and dedifferentiation, rather than the pink adipose tissue phenotype, as contributors to pro‐cancerous effects.

### 3.4 White Adipose Tissue

WAT is the most prevalent adipose tissue in humans and plays a central role in storing excess calories [8]. Structurally, WAT is composed mainly of white adipocytes, which are spherical in shape, contain a large single lipid droplet and have low levels of mitochondria [8, 12, 13]. WAT can be subcategorised into subcutaneous fat (SCAT), typically found beneath the skin, and visceral adipose tissue (VAT), around internal organs [60], with the latter being a more significant predictor of mortality than SCAT [61]. Visceral adipose tissue is primarily located in the mesentery and omentum, whereas SCAT, constituting approximately 80% of all body fat, is located in the femerogluteal regions, back and anterior abdominal wall [35]. Different fat depots display unique patterns of adipokine secretion, influencing metabolic and inflammatory states. Typically, SCAT contributes to a less intense inflammatory reaction than VAT [36], possibly due to lower production of IL‐6 [62].

On the other hand, VAT in obese rodent models contained an increased number of pro‐inflammatory immune cells, including T cells, B cells, macrophages, mast cells and neutrophils, as well as a decrease in anti‐inflammatory immune cells, such as natural killer T cells, Th2 T cells and T regulatory (Treg) cells [63]. Inflammation is amplified by pro‐inflammatory adipokines, which contribute to metabolic dysfunction and systemic inflammation [64], which is associated with an increased risk of cancer and poor cancer patient outcomes [65, 66]. This is reflected in studies in which higher VAT correlates with worse outcomes in patients with metastatic colorectal cancer [37], endometrial cancer [38, 39] and breast cancer [40]. However, high VAT previously predicted better survival in men but worse in women with rectal cancer [41]. These findings possibly highlight the broader role of VAT in metabolically unhealthy obesity, which in turn can promote breast cancer development (Figure 1) [63].

## 4 Adipose Tissue–Tumour Interaction

Each adipose tissue type and depot contribute uniquely to breast cancer progression, and their role becomes more pronounced when their direct proximity to tumours is considered. Adipocytes can interact directly with cancer cells. This was observed as an association between invasion of adipose tissue at the tumour margin and increased lymph node metastasis in patients with invasive breast carcinoma, regardless of their BMI [67].

The close proximity of adipocytes to cancer cells has been reported in numerous solid tumours during growth, invasion or metastasis, including prostate cancer [68], ovarian cancer [69], melanoma [70] and breast cancer [71]. Evidence from prostate cancer suggests that the direct interaction between cancer cells and adjacent fat tissue may drive cancer progression, even when systemic markers of obesity improve. For instance, periprostatic adipose tissue has been shown to support the directed migration of prostate cancer cells, highlighting a local pro‐tumourigenic effect independent of systemic factors [72]. In another study, weight reduction averaging 5.5% resulted in reduced visceral adipose tissue, insulin, total cholesterol, LDL cholesterol, leptin and leptin–adiponectin ratio. However, despite these systemic improvements, prostate tissue markers associated with cancer progression did not change significantly [73]. This suggests that the direct cancer–adipose tissue interaction may persist as a driver of cancer progression, irrespective of systemic inflammation or metabolic dysfunction.

Adipocytes play an active role in tumour invasion, particularly at the invasive tumour front, where they engage in direct interactions with cancer cells. Tumour cell–adipocyte interactions play a key role in shaping the TME, influencing cancer progression at multiple levels. Adipocytes, particularly those in close proximity to tumours, undergo phenotypic changes through lipolysis, allowing them to be converted to cancer‐associated adipocytes (CAAs) [74]. These cells actively contribute to tumourigenesis by secreting inflammatory adipokines, altering metabolic dynamics, and facilitating direct cellular interactions that enhance tumour aggressiveness. The parallels between cancer‐associated fibroblasts (CAFs) and CAAs are significant, as both originate from the mesenchymal stem cell lineage and support cancer progression [75]. In the TME, CAFs and CAAs can supply fatty acids directly to cancer cells to support their metabolic requirements. Additionally, lipid droplet formation in the TME, especially under hypoxic conditions, serves as an energy reservoir for cancer cells [76].

Hypoxia in tumours, especially in cells in the centre of the tumour [77], together with hypoxia that occurs as a consequence of the expansion of adipose tissue [78], results in elevated Hypoxia‐Inducible Factor 1 (HIF‐1). The elevation of HIF‐1 in turn drives the development of breast cancer through angiogenesis, glycolysis, immune escape and metastasis [79].

One study demonstrated that cancer cell invasion was significantly increased when co‐cultured with adipocytes. These adipocytes lose their lipid content under the influence of tumour cells. This lipid depletion coincided with the overexpression of pro‐inflammatory markers and a decrease in the expression of adipocyte markers, driving the transformation of adipocytes into fibroblast‐like cells with cancer‐associated properties. These cells were termed CAAs and promoted invasive behaviour [80]. Similarly, another study demonstrated that adipocytes lost their lipid content, accompanied by significantly lower expression of adipocyte‐related genes in a co‐culture model of adipocytes and cancer cells [81].

**Figure 2:**
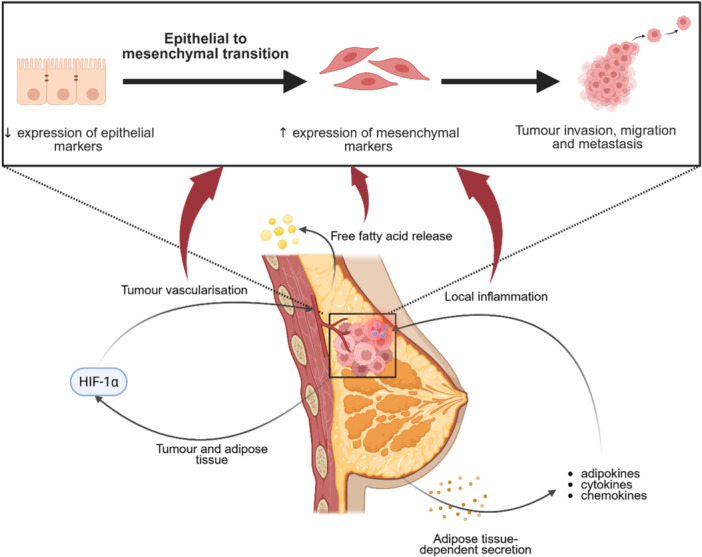
The direct adipose tissue–tumour interaction, driving multiple mechanisms in the TME. These mechanisms include (a) adipose tissue‐dependent secretion of adipokines, cytokines and chemokines, which contribute to a pro‐inflammatory microenvironment; (b) tumour and adipose tissue hypoxia, contributing to tumour vascularisation; and (c) adipose tissue‐associated release of FFAs, fuelling the metabolic requirements of tumours. Collectively, these mechanisms contribute to the EMT of breast cancer cells, leading to tumour invasion, migration and metastasis. This figure was created with BioRender.com.

During cancer progression, adipocytes near the tumour are thought to undergo dedifferentiation, first becoming CAAs and then transforming into fibroblast‐like cells [82]. A direct interaction between adipocytes and lung adenocarcinoma A549 cells has been observed to promote cancer cell proliferation, migration, epithelial–mesenchymal transition (EMT) and metabolic reprogramming in vitro. A549 cells co‐cultured with adipocytes showed increased glucose consumption, lactate production (indicative of the Warburg effect), and lipid droplet accumulation, along with decreased E‐cadherin and increased vimentin expression (indicative of EMT). Adipocytes exhibit reduced lipid droplet size and triglyceride content, releasing more free fatty acids (FFAs), which can be utilised by cancer cells to meet their metabolic needs [83]. Additionally, larger tumours or those that show lymph node involvement demonstrate higher levels of IL‐6 in peritumoralocytes. This could indicate the reciprocal relationship between cancer cells and neighbouring adipocytes, which in turn adopt a CAA phenotype, further contributing to tumour progression (Figure 2) [84].

## 5 Metabolic Remodelling

Direct adipocyte–tumour interactions drive metabolic remodelling in breast cancer cells [85]. Cancer cells exhibit remarkable plasticity in terms of metabolic adaptation, switching from one metabolic state to another to meet the energy and biosynthetic demands of rapidly proliferating tumour cells [76]. In the context of obesity, much emphasis has been placed on glucose and lipid metabolism.

### 5.1 Glucose Metabolism (Warburg Effect)

**Figure 3:**
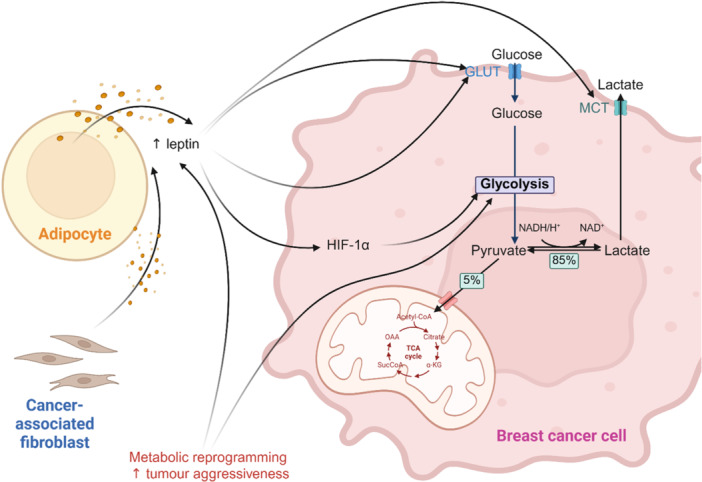
Obesity‐associated metabolic reprogramming in breast cancer cells in the context of the Warburg effect. This figure highlights the role of CAAs and CAFs in increasing leptin secretion within the TME. Elevated leptin levels associated with obesity promote breast cancer aggressiveness by (a) stabilising HIF‐1α, leading to enhanced glycolysis; (b) upregulating GLUT1, thereby increasing glucose uptake; and (c) increasing MCT4 expression, thereby facilitating enhanced lactate export. These alterations reduce mitochondrial oxidative phosphorylation and promote a glycolytic phenotype, creating a positive feedback loop that sustains high leptin levels, metabolic reprogramming, and tumour progression. Negative tumour‐promoting effects are indicated in red. This figure was created with BioRender.com.

The increased leptin/adiponectin ratio associated with obesity may favour tumour progression by contributing to the metabolic reprogramming of cancer cells (Figure 3) [86]. Rapidly proliferating cancer cells require increased energy and macromolecules [87], which they obtain through extensive metabolic reprogramming [15]. This metabolic reprogramming ensures that cancer cells can thrive in both hypoxic and normoxic environments, supporting rapid growth and metastasis while shaping the TME [88]. Metabolic reprogramming in cancer enables tumour cells to adapt to hypoxia in the TME by relying on glycolysis for energy. This process, known as the Warburg effect, generates lactate as a byproduct, which is used by tumour cells as an additional source of fuel, forming a metabolic symbiosis within the tumour [15].

Under hypoxic conditions, HIF‐1α enhances glucose transporter‐1 (GLUT‐1), which prevents hypoxia‐induced cell death [89]. The upregulation of GLUT‐1 ensures tumour survival by facilitating enhanced glucose uptake in tumour cells and is therefore also recognised as a hallmark of tumourigenesis [90]. In addition to HIF‐1α, insulin has been reported to upregulate GLUT‐1 expression [91]. One study concluded that the effects of leptin and INF‐γ on the growth and survival of breast cancer cells (MCF‐7 and MDA‐MB‐231) depend on GLUT‐1, as these effects were blocked when a GLUT‐1 inhibitor was introduced [92]. Another study reported that blocking GLUT‐1 reduced tumour growth and induced apoptosis [93]. Increased expression of GLUT‐1 has been associated with higher invasiveness of MCF‐7, MDA‐MB‐435 and MDA‐MB‐231 breast cancer cell lines [94]. In breast carcinoma patients, GLUT‐1 expression was found to be linked to poor prognosis [95]. Another study showed that GLUT‐1 was overexpressed in 60% of invasive breast carcinomas and that its overexpression was associated with more aggressive cancers [96].

Leptin promotes the upregulation of glucose uptake via GLUT‐1 and stimulates glycolysis by elevating the expression and activity of rate‐limiting enzymes in glycolytic pathways, such as pyruvate kinase M2 (PKM2), hexokinase and lactate dehydrogenase A (LDHA). These alterations were accompanied by enhanced processes like EMT, cell migration and invasion [86]. Additionally, leptin contributes to the stabilisation of HIF‐1α, a critical driver of the Warburg effect [86, 97], potentially through the suppression of p53. In turn, HIF‐1α activates glycolytic enzymes, such as LDHA, PKM2 and hexokinase [86]. In addition to regulating glycolysis, leptin also enhances other metabolic processes, including increased uncoupled respiration and elevated expression of the monocarboxylate transporter 4 (MCT4). The latter is a known lactate exporter, and its elevated expression leads to greater amounts of lactate available to cancer cells, which further promotes tumour growth and survival under hypoxic conditions [97].

Furthermore, leptin has emerged as a key player in mediating the interaction between tumour cells and CAFs, which form part of the tumour stroma. CAFs support tumour growth by secreting large amounts of pyruvate and lactate generated by aerobic glycolysis into the TME. In addition to contributing to the Warburg effect through the upregulation of glycolysis, CAFs secrete leptin and increase inflammation through Tumour Necrosis Factor alpha (TNF‐α) and IL‐6 secretion, while decreasing adiponectin. CAF‐derived leptin promotes the malignant behaviour of breast cancer cells, which in turn stimulates adjacent CAFs to reprogramme and produce even more leptin. Ultimately, this cycle amplifies leptin signalling and enhances cancer cell growth and invasiveness [86]. In addition to secreting leptin, CAFs express leptin receptors, creating an autocrine loop that further promotes cancer cell growth and invasion [97].

In contrast to leptin, adiponectin exerts opposing effects on breast cancer. Adiponectin counteracts tumourigenic effects driven by the Warburg effect by suppressing glycolysis, lactate production, and GLUT‐1 expression while promoting oxidative phosphorylation and limiting tumour growth [86]. A meta‐analysis of 27 articles showed that reduced serum adiponectin levels (as seen in obesity) are associated with an increased risk of breast cancer [98].

### 5.2 Lipid Metabolism

Although cancer is often investigated through the lens of glucose metabolism, lipid metabolism is worth investigating in the context of obesity‐driven cancer progression. In this context, aberrant lipid metabolism in cancer has received increasing attention because of its potential role in driving cancer metastasis and invasion.

A recent study showed that adipocytes incubated with conditioned media derived from explants of human breast adipose tissue from tumours secrete soluble factors into the medium. Through paracrine signalling, these factors induce the browning of adjacent adipocytes, lipolysis, and possible dedifferentiation. The latter was characterised by reduced expression of FABP4, PPARγ and CAV‐1, and increased expression of C/EBPβ‐LIP. Collectively, these processes (dedifferentiation, lipolysis and browning) may contribute to a tumour‐supportive microenvironment and serve as prognostic indicators for breast cancer. However, this study emphasises that future studies should clarify the direct role of these processes in tumour progression [99]. Another study investigating the effects of 3T3‐L1 adipocyte conditioned medium on MCF‐7 cells demonstrated that in the presence of abundant triglycerides (typically found in adjacent adipose tissue), MCF‐7 cells favour lipolysis and subsequently increase beta‐/fatty acid oxidation (FAO). Ultimately, this metabolic shift led to increased aggressiveness of breast cancer cells, characterised by increased cell viability, proliferation and migration. This metabolic shift led to a significant decrease in lactate generation (indicating a shift away from the Warburg effect), accompanied by an increase in acetate generation. The latter is a byproduct that can feed into the tricarboxylic acid cycle and produce energy via aerobic respiration. This adaptation marks a reversal of the classical Warburg effect, termed the ‘Warburg effect inversion’, wherein cancer cells, exposed to high levels of lipids, revert to oxidative metabolism rather than relying on aerobic glycolysis [100]. However, unlike the ‘reverse Warburg effect’, which depends on metabolic support from stromal cells in the TME [101], this inversion is driven intrinsically by cancer cells in response to high adiposity, independent of stromal cell contributions [100]. Similar metabolic reprogramming has previously been observed in ovarian cancer cells [69], suggesting that this may reflect a broader mechanism in obesity‐associated cancers.

Within the TME, tumour cells can also increase de novo lipogenesis, fatty acid uptake, and FAO to meet their energy requirements and support lipid accumulation [102]. Tumour cells shift towards FAO for energy, especially under hypoxic conditions, to promote their proliferation, survival, drug resistance and metastasis [103]. This shift is clinically significant, as increased lipid utilisation is a hallmark of aggressive carcinomas [104]. One study reported that direct contact of adipocytes with breast cancer cells led to increased adipocyte lipolysis. Increased lipolysis leads to the elevated release of FFAs that can be utilised by breast cancer cells. However, the specific signalling molecules released by breast cancer cells that drive lipolysis in adjacent adipocytes remain unknown [105]. Fatty acids, which provide nearly double the energy of glucose, become a primary fuel source through FAO, enabling tumour cells to sustain rapid proliferation [104]. Despite a sufficient dietary lipid supply, tumour cells also synthesise fatty acids de novo [104]. Activation of the de novo fatty acid synthesis pathway appears integral to carcinogenesis, as it supplies fatty acids critical for cell membrane construction and signalling [106]. In more aggressive cancers, extracellular fatty acid uptake is also utilised as endogenous lipogenesis becomes insufficient to sustain their metabolic requirements [104]. Certain cancers may utilise both lipogenic and lipolytic pathways to obtain fatty acids, thereby supporting continuous proliferation and survival [106].

**Figure 4:**
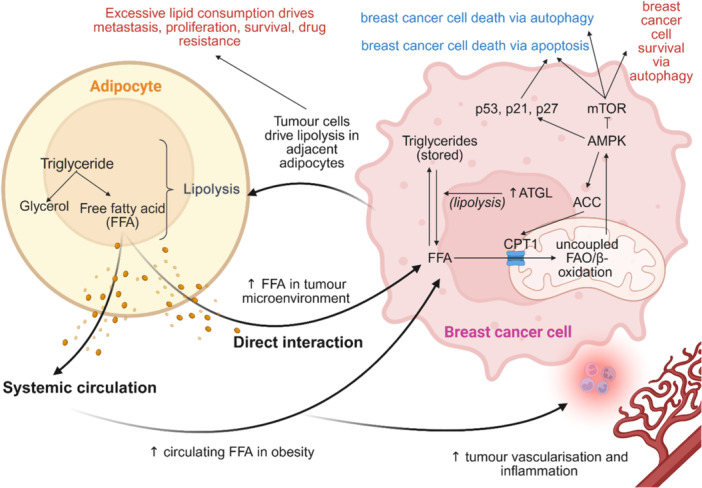
Obesity‐associated metabolic reprogramming in breast cancer cells in the context of lipid metabolism. The figure highlights how lipolysis leads to increased FFA levels in both the systemic circulation and TME, which are taken up by breast cancer cells and stored as triglycerides until needed. Increased ATGL levels in breast cancer cells drive lipolysis and the subsequent release of FFA, which enter the mitochondria through CPT1 and undergo uncoupled FAO. Low ATP production by FAO promotes AMPK activation, which may play a dual role in breast cancer cell survival. Additionally, AMPK suppresses ACC, allowing more FFA to be taken up into the mitochondria. Enhanced lipid consumption promotes breast cancer aggressiveness. Negative tumour‐promoting effects are indicated in red, while positive tumour‐suppressing effects are indicated in blue. This figure was created with BioRender.com.

In cases where adipose tissue and cancer are in direct contact with each other, FFAs released by adipocytes can be repurposed by tumour cells to fuel their rapid proliferation [107]. A study aimed at elucidating the impact of mature adipocytes on breast cancer cell behaviour demonstrated that co‐culture with adipocytes and exposure to adipocyte‐conditioned media promoted the proliferation and migration of MCF‐7 and MDA‐MB‐231 cells. In this context, the transfer of fatty acids between adipocytes and cancer cells plays a significant role in the breast cancer TME [108]. Another study, utilising breast cancer cell lines co‐cultured with adipocytes, showed that FFAs are released from lipid droplets through an adipose triglyceride lipase (ATGL)‐dependent lipolytic pathway. The released FFAs are utilised in FAO, which is active in cancer but not in normal breast epithelial cells. However, in co‐cultured cells, FFAs undergo uncoupled FAO upon entering the mitochondria via CPT1. Decreased ATP levels from FAO activate AMP‐activated protein kinase (AMPK), which plays a complex and dual role in cancer cell fate. First, AMPK may initiate cancer cell death through apoptosis by activating p53, p21 and p27 [109], and inhibiting mammalian target of rapamycin. The latter may also initiate autophagy [110], which may induce or prevent cell death in cancer cells, depending on the duration and intensity [111]. Second, AMPK drives lipid metabolism in breast cancer cells by suppressing acetyl‐CoA carboxylase (ACC), thereby allowing for more FFA uptake into the mitochondria [105]. Lipid metabolism in breast cancer cells is a driver of tumour aggressiveness by promoting metastasis, proliferation, survival and drug resistance (Figure 4) [105, 107].

## 6 Oxidative Stress

Multiple studies have highlighted the significant link between reactive oxygen species (ROS) accumulation and obesity, which leads to oxidative stress [112, 113, 114, 115, 116]. The latter is often accompanied by chronic inflammation, which has been shown to correlate with the onset of various types of cancer, including liver, colorectal, pancreatic, breast and lung cancer [117], possibly by altering the TME [118]. The persistent oxidative and intracellular stress associated with chronic inflammation also contributes to deoxyribonucleic acid (DNA) damage and the occurrence of DNA mutations, thereby driving cancer progression [117].

**Figure 5:**
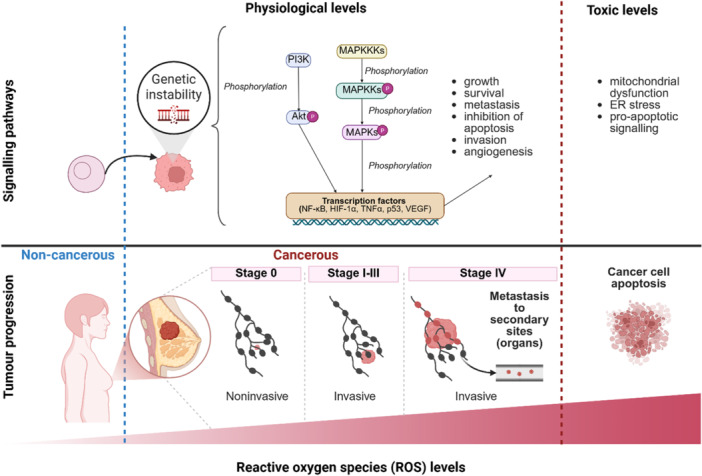
The role of ROS accumulation due to obesogenic conditions in breast cancer progression. At low levels, ROS remain within the physiological limits associated with a non‐cancerous state. Obesity contributes to increased ROS levels within the physiological range. Increasing ROS plays a role in contributing to genomic instability, thereby facilitating breast cancer initiation. With further elevation, ROS activate oncogenic signalling pathways, such as MAPK and PI3K/AKT, promoting cell proliferation, survival, metastasis, invasion, angiogenesis and resistance to apoptosis, thereby driving tumour aggressiveness. However, when ROS levels exceed the physiological threshold and reach toxic concentrations (usually induced by therapeutic intervention), opposing effects are exerted, such as mitochondrial dysfunction, endoplasmic reticulum (ER) stress, and pro‐apoptotic signalling, ultimately leading to cell death. This figure was created with BioRender.com.

ROS play a dual role in the body and influence breast cancer development and progression. While normal ROS levels support physiological functions, elevated levels (although still below toxicity thresholds) can damage mitochondrial DNA, activate proto‐oncogenes, and suppress tumour suppressor genes, contributing to breast cancer initiation [119]. In addition, high levels of ROS, often associated with obesity [120], contribute to cancer progression by promoting tumour growth and metastasis. This is achieved through elevated ROS levels, which denature lipids, proteins, and nucleic acids, thereby activating signalling pathways [121]. These include PI3K/Akt [121] and MAPK pathways (including ERK, JNK and p38) [119], promoting cell growth, survival, metastasis, inhibition of apoptosis, invasion and angiogenesis (Figure 5) [122].

In hypoxic environments (evident in both obesity and cancer), elevated HIF‐1 levels induce mitochondrial dysfunction and ROS production, leading to cell damage. To counteract this, selective mitochondrial autophagy (mitophagy) is activated to remove damaged mitochondria and reduce the ROS levels. HIF‐1 plays a key role in this process by promoting the transcription of BNIP3 and BNIP3L, which regulate autophagy and mitophagy [123]. In breast cancer tissues and cell lines, BNIP3 expression was upregulated and associated with cell proliferation, invasion, migration and autophagy under hypoxic conditions [124]. Additionally, TNBC cell lines showed a reliance on ROS for survival, as antioxidant treatment selectively induced cell death in TNBC cells, but not in oestrogen receptor‐positive (ER+) cells. The mitochondria were identified as the primary ROS source in TNBC cells [125]. Furthermore, ROS is known to contribute to metastasis by activating signalling pathways, such as NF‐κB and ERK/MAPK [126].

## 7 Conclusion and Future Perspectives

The molecular mechanisms underlying breast cancer progression in the context of obesity are complex and multifactorial. Obesity may alter the molecular landscape of breast cancer through direct and indirect mechanisms. The chronic, low‐grade inflammatory state, characteristic of obesity, may foster a pro‐tumourigenic milieu, with adipokines, chemokines, cytokines and immune cells working in concert with ROS. Collectively, these factors may activate multiple signalling pathways, including but not limited to NF‐κB, JNK, JAK/STAT3, PI3K and MAPK, to promote breast cancer cell proliferation, invasion, migration, survival, EMT and metabolic reprogramming. However, these effects are often distinct, depending on the adipose depot and the type of adipose tissue from which they are secreted. This warrants further investigation and tissue‐specific monitoring of these factors over time to elucidate their roles in breast cancer progression in the context of obesity. Deeper insights into these factors and immune profiles may offer unique opportunities for diagnosis and therapeutic interventions.

At the cellular level, obesity facilitates direct crosstalk between adipocytes and adjacent breast cancer cells. These interactions promote an immunosuppressive TME and metabolic adaptation, possibly switching between glucose metabolism (Warburg effect) and lipid metabolism (beta‐oxidation), to promote breast cancer progression. The metabolic plasticity and immune profile of breast cancer, which may change in the context of obesity, make it difficult to predict outcomes, especially for the different subtypes and stages of breast cancer. As such, these mechanisms and pathways should be explored thoroughly, not only in various subtypes but also longitudinally, to understand how this profile might change depending on the breast cancer stage. By utilising these insights, we can capitalise on our understanding of these metabolic mechanisms in terms of diagnosis (using PET tracers specific to glycolysis and beta‐oxidation) and targeted treatment.

Currently, research exploring how molecular mechanisms evolve over time to influence the initiation, progression and metastasis of breast cancer is lacking. This gap largely stems from ethical constraints, as it is not feasible to delay clinical intervention in humans for the sake of longitudinal observation, making it challenging to study these processes in real time during disease development. Consequently, most research on this topic involves two‐dimensional or co‐culture models, which do not fully capture the complex and dynamic inter‐organ interactions with the TME. Therefore, longitudinal in vivo approaches could offer critical insights into how obesity‐induced breast cancer progression affects various organ systems.

Despite the proposal of multiple molecular theories, it is essential to recognise that obesity‐driven breast cancer progression cannot be entirely understood through isolated mechanisms or viewed as separate theories. Instead, it should be viewed as a complex, overlapping network of signalling molecules, metabolic shifts and immune cells that interact collectively to promote breast cancer progression. This interconnectedness highlights the need for comprehensive research that simultaneously examines multiple mechanisms within the same subject to understand how they interact with or regulate each other. In addition to offering insights into possible therapeutic targets, this study may also inform whether combination treatment should be considered. Understanding the underlying mechanisms that drive breast cancer in the context of obesity may offer a promising path towards the development of more effective management strategies in the face of rising global obesity and cancer rates.

## Author Contributions

Charlise Basson wrote the original draft with critical review and input from Melvin A. Ambele, Michael S. Pepper and Anna M. Joubert. All authors contributed to the study's conception and design. All authors have given their final approval for the manuscript.

## Conflicts of Interest

The authors declare no conflicts of interest.

## Data Availability

Data sharing is not applicable to this article as no data sets were generated or analysed during the current study.
